# Survival advantage of cytoreductive surgery and hyperthermic intraperitoneal chemotherapy (HIPEC) for advanced gastric cancer: experience from a Western tertiary referral center

**DOI:** 10.1007/s00423-021-02102-2

**Published:** 2021-03-11

**Authors:** Fausto Rosa, Federica Galiandro, Riccardo Ricci, Dario Di Miceli, Fabio Longo, Giuseppe Quero, Antonio Pio Tortorelli, Sergio Alfieri

**Affiliations:** 1 Department of Digestive Surgery, Fondazione Policlinico Universitario Agostino Gemelli IRCCS, Largo A. Gemelli, 8, 00168 Rome, Italy; 2 Università Cattolica del Sacro Cuore, Rome, Italy; 3 General Surgery, Ospedale Buccheri La Ferla, Palermo, Italy; 4 Department of Pathology, Fondazione Policlinico Universitario Agostino Gemelli IRCCS, Rome, Italy

**Keywords:** Gastric cancer, HIPEC, Peritoneal carcinomatosis, Surgery

## Abstract

**Background:**

Selection criteria and prognostic factors for patients with advanced gastric cancer (AGC) undergoing cytoreductive surgery (CRS) plus hyperthermic intra-operative peritoneal chemotherapy (HIPEC) have not been well defined, and the literature data are not homogeneous. The aim of this study was to compare prognostic factors influencing overall (OS) and disease-free survival (DFS) in a population of patients affected by AGC with surgery alone and surgery plus HIPEC, both with curative (PCI, peritoneal carcinomatosis index > 1) and prophylactic (PCI = 0) intent.

**Methods:**

A retrospective analysis of a prospectively collected database was conducted in patients affected by AGC from January 2006 to December 2015. Uni- and multivariate analyses of prognostic factors were performed.

**Results:**

A total of 85 patients with AGC were analyzed. A 5-year OS for surgery alone, CRS plus curative HIPEC, and surgery plus prophylactic HIPEC groups was 9%, 27% and 33%, respectively. Statistical significance was reached comparing both prophylactic HIPEC vs surgery alone group (*p* = 0.05), curative HIPEC vs surgery alone group (*p* = 0.03), and curative vs prophylactic HIPEC (*p* = 0.04). A 5-year DFS for surgery alone, CRS + curative HIPEC, and surgery + prophylactic HIPEC groups was 9%, 20%, and 30%, respectively. Statistical significance was reached comparing both prophylactic HIPEC vs surgery alone group (*p* < 0.0001), curative HIPEC vs surgery alone group (*p* = 0.008), and curative vs prophylactic HIPEC (*p* = 0.05).

**Conclusions:**

Patients with AGC undergoing surgery plus HIPEC had a better OS and DFS with respect to patients treated with surgery alone.

## Introduction

Gastric cancer is the sixth most prevalent malignant tumor worldwide and the third leading cause of cancer-related death. The International Agency for Research on Cancer estimated that there were about one million new cases of gastric cancer and 782,685 deaths from gastric cancer in 2018 [1]. Many patients in the western world with AGC die from metastases [2].

The peritoneal cavity is also a frequent site for metastatic disease after resection, particularly in patients with serosa-infiltrating tumors [3, 4]. Patients with AGC and peritoneal carcinomatosis (PC) have a poor prognosis, with a median survival of 3.1 months without treatment [5]. Systemic chemotherapy extended the median survival time to 11 months in patients with AGC compared with best supportive care alone [6].

Extended resection involving gastrectomy and peritonectomy combined with administration of HIPEC may improve survival in patients with PC [7–9]. HIPEC possesses a theoretical advantage over systemic treatment delivering high drug concentrations directly to the peritoneal cavity, resulting in a reduced systemic toxicity [10–12]. In addition, high drug concentrations are achieved in the portal vein [13, 14].

Extended survival with HIPEC in AGC has been demonstrated, but the lack of standardized protocols has led to difficulties comparing and interpreting results [15]. A meta-analysis demonstrated improved overall survival with HIPEC with or without early postoperative intraperitoneal chemotherapy [16].

Perhaps the most appropriate use of HIPEC in AGC would be prophylactic, suggesting an adjunct to curative surgical resection in patients with a high risk of peritoneal recurrence. Not surprisingly, the majority of data related to HIPEC in AGC is prophylactic against peritoneal recurrences. The theoretical rationale and synergistic effect is that large diluent volumes in HIPEC wash out most of the intraperitoneal free cancer cells and chemotherapy destroys remaining cancer cells [17].

With the aim of contributing to this issue, we have conducted a comparative observational analysis between patients undergoing CRS alone and those who received gastrectomy plus HIPEC both with curative (PCI > 1) and prophylactic (PCI = 0) intent.

## Methods

A retrospective analysis of prospectively collected data was conducted regarding patients with AGC observed and treated at the Digestive Surgery Unit, Fondazione Policlinico Universitario “A. Gemelli” IRCCS, from January 2006 to December 2015.

We preliminarily obtained Institutional Review Board approval to use patient data.

Patients analyzed were divided into the following 3 groups:

- Surgery plus HIPEC with curative intent: AGC patients with apparent peritoneal dissemination who underwent cytoreductive surgery, including gastrectomy and partial peritonectomy of peritoneal sections affected by implants, followed by HIPEC
- Surgery *plus* HIPEC with prophylactic intent: AGC patients with serosa invasion and consequent high risk of intraperitoneal progression, who underwent gastrectomy followed by HIPEC
- Surgery alone: AGC patients who underwent only gastrectomy due to the presence of exclusion criteria for HIPEC

The same team of oncologists performed all surgeries, and all patients had to provide a written informed consent before the intervention.

Patients were divided according to the type of surgical procedure performed.

### Inclusion/exclusion criteria

All patients were submitted to a complete clinical evaluation, including laboratory tests, with complete blood cell count and serum chemistry.

In order to exclude extra-abdominal disease and to assess the possibility of optimal cytoreduction, all patients underwent to a CT scan or FDG-PET/CT scan. A preoperative laparoscopy was selectively performed for the purpose of selecting patients for neoadjuvant therapy.

Patients with histologically documented AGC, with a preoperative stage II to IV, with peritoneal carcinomatosis (stage IV) or at high risk to develop it due to serosal involvement, were included in the study.

Inclusion criteria were as follows: age 18–80 years, normal cardiac, respiratory, liver and renal functions, and no hematological alterations.

Exclusion criteria for HIPEC were uncontrolled severe infection and/or medical problems unrelated to malignancy which would limit full compliance with the protocol or expose the patient to extreme risk of life.

All patients in surgery alone group were excluded from HIPEC due to the presence of an exclusion criteria.

All patients included were analyzed without defining any cut-off value for PCI and CC score.

We recorded hospital morbidity and mortality, type of treatment, histologic type according to Lauren [18], and demographic characteristics, tumor size, and tumor location. The disease was staged according to the 8th Edition of the American Joint Committee on Cancer and the International Union Against Cancer Staging System (UICC) [19, 20].

### Surgical rules

Based on categories established by the Japanese Gastric Cancer Association [21], the regional extent of nodal involvement after radical procedures was also recorded.

At the end of the operation, the surgeon resected all lymph nodes from the surgical specimen and identified their distribution and tumor location according to the classification by the Japanese Gastric Cancer Association [21].

The PCI score was calculated at laparotomy [22]. The CC score was calculated for all patients in the three groups. CC-0 reflected no remaining visible disease. CC-1, 2, and 3 implied remaining disease less than 2.5 mm, 2.5 to 2.5 cm, and greater than 2.5 cm [22].

After total gastrectomy with D2 lymph node dissection, esophagojejunostomy (using a circular stapler, diameter 25 mm) was used routinely for Roux-en-Y reconstruction.

In case of subtotal gastrectomy, intestinal continuity was restored by means of Billroth II or Roux-en-Y gastrojejunostomy, at discretion of the surgeon.

In case of carcinomatosis, CRS was performed removing all peritoneum and visceral organs involved.

Extensive surgery (associated resections) because of suspicion of direct tumor invasion or carcinomatosis was defined as combined resection of adjacent organs (spleen, left pancreas, liver, colon, adrenal gland, diaphragm, abdominal wall, and small intestine).

### HIPEC

HIPEC was carried out according to the coliseum technique [22]. Two inflow and two outflow 29 French catheters were placed in the upper and lower abdominal quadrants, respectively. The HIPEC procedure was administered for 90 min with an inflow temperature of 41–42 °C and an outflow temperature of 39–40 °C, using mitomycin C (MMC) at a dose of 15 mg/m^2^ and cisplatin at a dose of 75 mg/m^2^. As perfusate volume, a 2 L/m^2^ 0.9% NaCl solution was used. At the end of the procedure, an abdominal washout was performed with 3 L of crystalloid solution. After 90 min of perfusion, the abdomen was cautiously re-explored to control the hemostasis.

The temperature was monitored using digital probes placed in abdominal cavity at circuit level.

### Pathological data

Based on definitive pathologic findings, the potentially curative operations were classified as radical (R0-microscopic tumor free) or as R1-microscopic residual disease—according to the presence or absence of residual tumor. Palliative resection was classified based on R2 macroscopic

disease left behind. Frozen sections were not routinely used in the evaluation of margins, but only in the suspicion of a possible tumor infiltration.

### Postoperative course

The patients were monitored for 30-day postoperative complications and mortality.

Early postoperative complications were considered occurring within 30 days from surgery and with a severity grade 2 or more according to the Clavien-Dindo classification [23]. All postoperative complications were registered in the database during hospitalization or at the first follow-up, by telephone contact, within 30 days from surgery.

Postoperative mortality was defined as death within 30 days from surgery.

Perioperative chemotherapy was administered, in the majority of cases, according to the MRC Adjuvant Gastric Infusional Chemotherapy (MAGIC) protocol [24].

The oncologists decided about adjuvant chemotherapy administration, as previously reported [25], resulting in heterogeneity regarding chemotherapy, treatment protocols, and a number of cycles performed.

All patients included in the study were regularly followed up with a standardized protocol [26].

### Statistical analyses

All clinical and pathological data were prospectively stored in a GC database and evaluated for this study. All variables are expressed as the mean ± standard deviation (±), median, and interquartile range (IQR) when appropriate. The statistical significance of the difference between mean values was evaluated using the Student’s *t* test. All tests were two tailed. Categorical variables were assessed by the Pearson’s Chi-square test. Multivariable analysis was undertaken using the Cox proportional hazards model. The survival adjusted for censoring was calculated using the Kaplan-Meier method, and the medians were compared using the log-rank test. A *p* value < 0.05 was considered statistically significant.

All data were analyzed by SPSS version 25® (IBM, IL, USA).

## Results

During the study period, a total of 427 patients with GC underwent surgery with curative intent at the Digestive Surgery Unit of the Fondazione Policlinico Universitario “A. Gemelli” IRCCS of Rome.

Among them, 85 patients with advanced GC were retrospectively analyzed for this observational study. More specifically, forty-six patients (F/M ratio 25/21; mean age 55 years, range 28–76) underwent surgery plus HIPEC. In 50% (23/46) of cases, indication for HIPEC was a T3/T4 gastric cancer without peritoneal carcinomatosis (PCI = 0). Thirty-nine patients received CRS alone.

Clinico-demographic characteristics of all patients are shown in Table 1.

**Table 1:** Clinico-demographic characteristics of all patients

Patients, *n*	85
Age, years, mean (± SD)	61 ± 15.1
Female, *n* (%)	44 (52)
Primary tumor location	
Lower third, *n* (%)	36 (42)
Middle third, *n* (%)	34 (40)
Upper third, *n* (%)	15 (18)
Neoadjuvant therapy	
Chemotherapy, *n* (%)	38 (44.7)
No. of cycles, mean (+ SD)	5 ± 4.8
Chemoradiotherapy, *n* (%)	2 (2)
Response to treatment, *n* (%)	19 (50)
ASA**,** *n* (%)	
1	18 (21)
2	50 (59)
3	17 (20)
Indication for HIPEC	
Prophylactic (PCI = 0), *n* (%)	23 (27)
Curative (PCI ≥ 1), *n* (%)	23 (27)
No HIPEC, *n* (%)	39 (46)
Total harvested lymph nodes	
*n* < 15 (%)	8 (9.4)
*n* ≥ 15 (%)	77 (90.6)
Positive lymph nodes	
N0 (%)	10 (11.8)
N+ (%)	75 (88.2)
Adjuvant therapy	41 (48.2)
Length of stay, days, mean (+ SD)	13.4 + 9.3
Operation time**,** minutes, mean (+ SD)	338 + 92.7
Follow-up, months, median (IQR)	68

The median follow-up (IQR) was 68 months.

Excluding 4 patients lost during the study period and 3 patients who died during the postoperative hospital stay (1 in the curative HIPEC group and 2 in the only surgery group), follow-up was completed in 78 cases (91.7%). At the last evaluation, 54 (63.5%) patients had died.

Positive cytology was present only in 6 patients (26%) who underwent prophylactic HIPEC.

Thirty-eight patients (44.7%) received neoadjuvant chemotherapy with a pathological response in 19 cases (50%).

The majority of patients was preoperatively classified as ASA 2 (50 patients, 59%).

Seventy-seven patients (90.6%) had ≥ 15 lymph nodes retrieved, and 75 (88.2%) were N+.

The mean duration of surgical procedures was 338 (± 92.7) minutes, and the mean length of postoperative hospital stay was 13.4 (± 9.3) days.

Forty-one patients (48.2%) received adjuvant chemotherapy.

Clinico-demographic characteristics of the three groups are shown in Table 2.

**Table 2:** Clinico-demographic characteristics of the three study groups

	Prophylactic HIPEC (*n* = 23)	Curative HIPEC (*n* = 23)	No HIPEC (*n* = 39)	p*
Age, years, mean (+)	58 (35–74)	52 (28–76)	68 (41–86)	< 0.0001
Sex, *n* (%)				
Male	11 (48)	10 (43)	20 (51)	0.83
Female	12 (52)	13 (57)	19 (49)	
ASA score, *n* (%)				
ASA I	4 (17)	10 (43)	2 (5.1)	0.04
ASA II	14 (61)	10 (43)	12 (30.8)	
ASA III	5 (22)	3 (13)	25 (64.1)	
Tumor location, *n* (%)				
Lower third	8 (35)	6 (26)	22 (56)	0.08
Middle third	12 (52)	10 (43)	12 (31)	
Upper third	3 (13)	7 (31)	5 (13)	
Neoadjuvant therapy	12 (52)	11 (47.8)	15 (38.5)	0.46
pTNM stage**,** *n* (%)				
IIB	3 (13)	0	4 (10.3)	0.008
IIIA	7 (30)	0	11 (28.2)	
IIIB	2 (9)	0	10 (25.6)	
IIIC	11 (48)	0	4 (10.3)	
IV	0	23 (100)	10 (25.6)	
PCI** range, *n* (%)				
0	23 (100)	0	29 (74.4)	< 0.0001
1–6	0	14 (61)	4 (10.2)	
7–15	0	7 (30)	6 (15.4)	
16–39	0	2 (9)	0	
CC score***, *n* (%)				
CC 0	23 (100)	19 (82.6)	32 (82.1)	0.003
CC 1	0	4 (17.4)	2 (5.1)	
CC 2	0	0	2 (5.1)	
CC 3	0	0	3 (7.7)	
R status, *n* (%)				
R0	22 (96)	18 (78.3)	32 (82.1)	0.03
R1	1 (4)	5 (21.7)	2 (5.1)	
R2	0	0	5 (12.8)	
Lauren classification, *n* (%)				
Diffuse type	13 (57)	12 (52)	19 (49)	0.98
Intestinal type	6 (26)	7 (30)	13 (33)	
Mixed type	4 (17)	4 (18)	7 (18)	
Total harvested lymph nodes				
*n* < 15 (%)	1 (4)	2 (8)	5 (12)	0.53
*n* > 15 (%)	22 (96)	21 (92)	34 (88)	
Positive lymph nodes				
N0 (%)	1 (4)	3 (13)	6 (15)	0.41
N+ (%)	22 (96)	20 (87)	33 (85)	
Adjuvant therapy	18 (78.3)	15 (65.2)	8 (20.5)	0.52

*Two-tailed Pearson’s Chi-square test

**PCI peritoneal carcinomatosis index

***CC score cytoreduction completeness score

A significant difference among the three groups was noticed regarding the distribution of ASA score, tumor location and tumor stage, PCI range, CC score, and *R* status.

Intra-operative and short-term outcomes for the three groups are shown in Table 3.

**Table 3:** Intra-operative and short-term outcomes.

	Prophylactic HIPEC (*n* = 23)	Curative HIPEC (*n* = 23)	No HIPEC (*n* = 39)	p*
Type of resection				
Total gastrectomy, *n* (%)	12 (52)	16 (69)	18 (46)	0.19
Subtotal gastrectomy, *n* (%)	11 (48)	7 (31)	21 (54)	
Associated resections, *n* (%)	8 (35)	15 (65)	10 (25)	0.008
Operation time, min, mean (± SD)	380 + 35.6	482 + 42.1	227 + 28.7	< 0.0001
EBL^**^, ml, mean (± SD)	204 ± 103.1	250 ± 153.0	190 ± 80.2	0.23
Postoperative complications**,** *n* (%)	9 (39)	9 (39)	18 (46)	0.8
Surgical complications**,** *n* (%)	4 (17)	7 (30)	7 (18)	0.44
Evisceration	0	1 (4)	1 (2)	
Intra-abdominal abscess	2 (8)	0	2 (5)	
Anastomotic leakage	1 (4)	1 (4)	4 (10)	
Bowel obstruction	0	1 (4)	0	
Bleeding	0	1 (4)	0	
Delayed gastric emptying	0	1 (4)	0	
Intestinal ischemia	0	1 (4)	0	
Wound Infection	1 (4)	1 (4)	0	
Medical complications, *n* (%)^†^	6 (26)	4 (17)	15 (38)	0.19
Clavien-Dindo **≥** 2, *n* (%)	3 (13)	6 (26)	9 (23)	0.51
Reoperation, *n* (%)	2 (8)	5 (21)	3 (7)	0.21
Length of stay**,** days, mean (+ SD)	11 ± 5.2	16 ± 3.7	16 ± 4.1	0.06
Postoperative mortality^χ^, *n* (%)	0 (0)	1 (4)	2 (5)	0.55

*Two-tailed Pearson’s Chi-square test

^**^EBL estimated blood loss

^**†**^Fever without signs of infection or need of antibiotics, hypertension, electrolyte imbalance, pulmonary atelectasis requiring physiotherapy, and transient confusion not requiring therapy

^χ^Death within 30 days from surgery

Among the three groups, a significant difference was detected as far as associated resections and operation time were concerned (*p* = 0.008 and *p* < 0.0001, respectively).

No differences between the three groups neither in terms of postoperative complications (*p* = 0.8) nor in terms of postoperative mortality (*p* = 0.55) rates were observed.

In the groups of patients who received HIPEC, only one case of postoperative intestinal ischemia, and one episode of acute renal failure was observed, probably HIPEC-related.

Prognostic factors affecting OS and DFS according to univariate analysis are shown in Table 4.

**Table 4:** Prognostic factors affecting OS and DFS according to univariate analysis in 85 patients with advanced GC

	Overall survival		Disease-free survival	
	%	p*	*n* (%)	p*
Age				
≤ 65	26.1	0.14	20.9	0.23
> 65	37.1		31.4	
Gender				
M	26.8	0.23	22.3	0.48
F	38.2		32.5	
ASA score				
≤ 2	35.7	0.44	27.8	0.23
> 2	26.7		22.3	
Tumor location				
Lower third	29.3	0.01	22.4	0.02
Middle/upper third	24.6		19.7	
Neoadjuvant therapy				
Yes	32.8	0.32	27.6	0.25
No	25.7		21.3	
TNM				
< IIIB	33.3	0.02	22.5	0.01
≥ IIIB	8		7.7	
PCI**				
< 6	31.7	0.01	25.7	< 0.0001
≥ 6	9.3		8.2	
CC score**				
0	30.9	0.009	28.9	< 0.0001
> 0	9.3		8.6	
R status				
R0	35.7	0.15	29.4	< 0.0001
R1/2	26.2		12.9	
Lauren type				
Diffuse	19.5	0.34	13.7	0.25
Others	26.4		22.3	
N. lymph nodes				
< 15	27.5	0.67	18.5	0.51
≥ 15	35.7		21.1	
Positive lymph nodes				
N0	33.6	0.04	29.5	0.05
N+	16.7		15.6	
Type of resection				
Total gastrectomy	21.8	0.01	20.1	0.007
Subtotal distal gastrectomy	36.4		29.3	
Associated resection				
No	30.9	0.16	27.9	0.23
Yes	19.5		17.4	
Operative time, min				
< 320	32.1	0.17	26.9	0.2
≥ 320	21.4		16.7	
Postoperative complications				
Yes	17.6	0.38	15.9	0.66
No	31.7		28.7	
HIPEC				
Yes	30	0.04	25	0.02
No	9		9	
HIPEC				
Prophylactic	33	0.04	30	0.05
Curative	27		20	
Adjuvant therapy				
Yes	31.2	0.71	28.2	0.42
No	26.4		23.6	

*Log-rank test

**PCI peritoneal carcinomatosis index

***CC score cytoreduction completeness score

Tumor location, stage IIIB, PCI ≥ 6, CC score > 0, N+, type of resection, HIPEC, and the type of HIPEC (prophylactic vs curative) significantly affected both OS and DFS. R status significantly affected only DFS (*p* < 0.0001).

Table 5 shows multivariate analysis of factors associated with OS and DFS.

**Table 5:** Prognostic factors affecting OS and DFS according to multivariate Cox regression in 85 patients with advanced GC

Variables	5-year OS		
	OR	95% CI	*p*
Medium/upper tumor location	1.7	0.95–3.11	0.07
TNM ≥ IIIB	1.49	0.81–2.72	0.19
PCI ≥ 6	1.76	0.77–4.09	0.005
CC ≥ 0	1.65	0.46–2.43	0.02
N+	1.92	0.73–5.03	0.001
Total gastrectomy	0.73	0.4–1.34	0.32
No HIPEC	1.47	1.23–2.99	0.05
	5-year DFS		
	OR	95% CI	*p*
Middle/upper third tumor location	1.4	0.78–2.5	0.25
TNM ≥ IIIB	1.49	0.83–2.69	0.18
PCI ≥ 6	2.65	1.23–5.74	0.013
CC ≥ 0	2.36	0.51–10.92	0.012
R +	2.78	1.18–3.37	0.03
N+	1.75	0.7–4.37	0.22
Total gastrectomy	0.76	0.41–1.41	0.39
No HIPEC	2.52	0.26–1.04	0.005

At the multivariate analysis for OS, PCI ≥ 6, CC > 0, N+ status, and the absence of HIPEC were statistically significant.

On the other hand, at the multivariate analysis, DFS was significantly influenced by PCI ≥ 6, CC > 0, R status, and the absence of HIPEC.

**Fig. 1:**
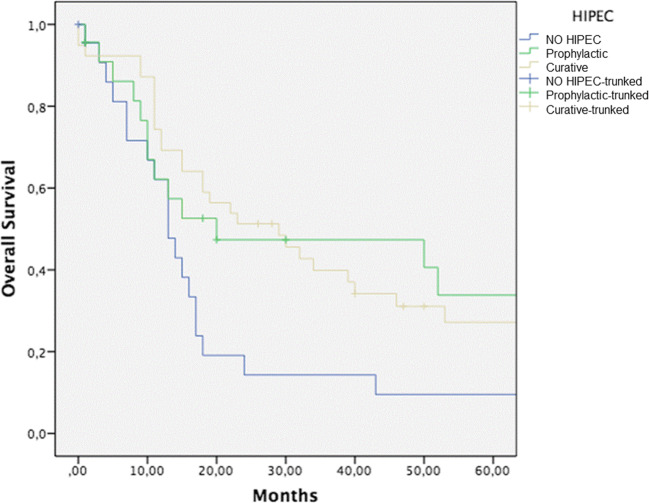
A 5-year OS for CRS alone, CRS plus curative HIPEC, and CRS plus prophylactic HIPEC groups

A 5-year OS for surgery alone, CRS + curative HIPEC, and surgery + prophylactic HIPEC groups was 9%, 27%, and 33%, respectively (Fig. 1). Statistical significance was reached comparing both prophylactic HIPEC vs surgery alone group (*p* = 0.05) and curative HIPEC vs surgery alone group (*p* = 0.03).

**Fig. 2:**
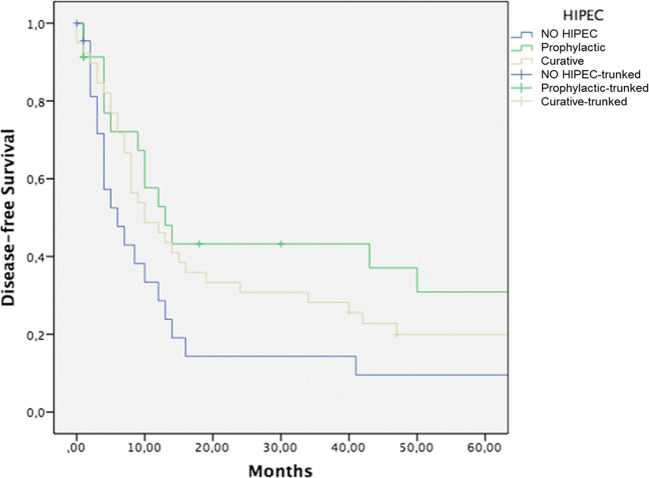
A 5-year DFS for CRS alone, CRS plus curative HIPEC, and CRS plus prophylactic HIPEC groups

Forty-six patients (54.1%) experienced a cancer recurrence, 23 in surgery alone group, 13 in curative HIPEC group, and 10 in prophylactic HIPEC group. In all cases, it was a peritoneal dissemination. A 5-year DFS for surgery alone, CRS plus curative HIPEC, and surgery plus prophylactic HIPEC groups was 9%, 20%, and 30%, respectively (Fig. 2) (*p* = ns). Statistical significance was reached comparing prophylactic HIPEC vs CRS alone group (*p* = 0.008).

The intraperitoneal recurrence rates in patients in surgery *plus* HIPEC with curative intent group, surgery in surgery plus HIPEC with prophylactic intent group, and in surgery alone group were 28.2%, 21.7%, and 65.4%, respectively (*p* = 0.007).

## Discussion

Despite the high level of evidence, data supporting the use of CRS + HIPEC for treating AGC, with or without PC, is still not accepted as a standard treatment, likely because AGC is still associated with a poor prognosis, even without peritoneal disease [27, 28].

Intraperitoneal administration of chemotherapy results in a regional dose intensification, i.e*.*, a high

intraperitoneal concentration of the drug with a low plasma concentration [29].

Scaringi et al. [30] reported that complete CRS plus HIPEC increased advanced AGC patients’ survival rates, especially in those without macroscopic peritoneal residuals.

However, wide application of CRS plus HIPEC is hampered by the adverse effects of chemotherapy.

To the best of our knowledge, our study represents the first experience comparing CRS alone, CRS plus HIPEC with curative intent, and CRS plus HIPEC with prophylactic intent in patients with AGC.

In our paper, we demonstrated that tumor location, advanced T stage, PCI > 6, CC score > 0, N+, type of resection, and the use of HIPEC significantly affected both OS and DFS. R status significantly affected only DFS (*p* < 0.0001).The earliest report of the use of HIPEC as an adjuvant treatment to prevent peritoneal recurrence was by Koga et al. [31]. They reported two studies, the first a historical study comparing 38 GC patients with serosal invasion who underwent curative surgery followed by HIPEC using MMC with a control group of 55 patients who underwent curative surgery without HIPEC. They found that the HIPEC group had a significantly improved 3-year survival (74% vs 53%, *p <* 0.04) with fewer peritoneal recurrences (36% vs 50%) respectively. Subsequently, they performed a randomized study in which patients were randomized to undergo curative surgery with HIPEC or only surgery. In this study also, they found that patients who received HIPEC had a trend towards a better 30-month survival compared to the control group (83% vs 67%) although this was not statistically significant.

Fujimoto et al. [32] reported a prospective study of 59 patients, 32 of whom had advanced AGC without PC who underwent curative surgery. The 2-year survival of the 10 patients who received HIPEC was significantly higher than that of the 20 patients who did not (56.5% vs 12.9%, *p =* 0.01). While no patient in the former group developed peritoneal recurrence, 8 patients in the latter group died due to peritoneal recurrence.

There have been various randomized controlled trials comparing HIPEC vs no HIPEC in patients with locally AGC who underwent a potentially curative resection [17]. Although there is some heterogeneity in these trials with respect to the drugs used, their dosage, duration of HIPEC, temperature achieved, etc., these trials provide level 1 evidence of the ability of adjuvant HIPEC to reduce peritoneal recurrence and improve survival. Not many studies have evaluated the effects of prophylactic HIPEC in patients with Cy+/P0 GC [33].

In a meta-analysis of 10 RCTs, Sun et al. [34] demonstrated a significant advantage in survival with the use of HIPEC, regardless of the chemotherapy used (MMC or 5-FU) and also regardless of whether adjuvant systemic chemotherapy was used or not.

In a pooled analysis of 16 RCTs, Mi et al. [28] reported a significant improvement in the 1, 2, 3, 5, and 9-year survival and a reduction in the peritoneal recurrence rates at 2, 3, and 5 years in patients who received HIPEC compared to those who did not.

These results indicate that intraperitoneal chemotherapy is best delivered at the time of surgery to treat the microscopic dissemination that occurs before or during surgery [28] and that hyperthermia has a synergistic effect with intraperitoneal chemotherapy perfusion.

In summary, adjuvant HIPEC used as prophylaxis against peritoneal recurrence in patients with high-risk GC (serosal invasion or nodal metastasis) is safe, significantly improves the survival, and reduces the risk of peritoneal recurrence. However, most of these RCTs have been conducted in Asian countries, and the data from the western world is scarce.

The GASTRICHIP study is a phase III randomized European multicenter study evaluating the role

of HIPEC with oxaliplatin in patients with GC who have either serosal infiltration and/or lymph nodal involvement and/or positive peritoneal cytology treated by a curative gastrectomy [35]. The most recent meta-analysis by Desiderio et al. [36] demonstrated a survival advantage of the use of HIPEC as a prophylactic strategy and suggests that patients whose disease burden is limited to positive cytology and limited nodal involvement may benefit the most from HIPEC. Moreover, for patients with extensive carcinomatosis, the completeness of cytoreductive surgery is a critical prognostic factor for survival [37]. Future RCTs should better define patient selection criteria.

The first report from the western world on role of extensive surgery plus HIPEC came from Sayag-Beaujard et al. [38]. The authors reported a phase II study of 42 patients with AGC with peritoneal disease who underwent HIPEC with MMC. The overall median survival was 10.3 months, and the 5-year survival was 8%.

In a large series of 107 patients reported in 2005, Yonemura et al. [39] compared 65 patients who underwent conventional surgery followed by HIPEC for PC from GC with 42 patients who had a peritonectomy as described by Sugarbaker [7] followed by HIPEC. The median survival for all 107 patients was 11.5 months, and the 5-year survival was 6.7%, but the 5-year survival for the patients who underwent peritonectomy and HIPEC was 27%. Performing a peritonectomy enabled a higher rate of complete cytoreduction and, subsequently, a better survival.

Compared to the most recent literature experiences, our study presented better 5-year-OS rates both for curative and prophylactic HIPEC (27% and 33%, respectively) comparing to CRS alone group (9%). Also, 5-year-DFS rates resulted significantly higher in patients undergoing HIPEC with respect to those who did not (20% and 30% vs 9%, respectively).

The French CYTO-CHIP study by Bonnot et al. [40] is the most recent multicentric study from 19 centers of the FREGAT and the BIG-RENAPE networks that focused especially on the effect of HIPEC after complete CRS using a propensity score analysis. With 277 patients, it represents actually the largest study concerning CRS-HIPEC and gastric cancer. It showed a strong positive effect of HIPEC after CRS versus CRS alone without additional morbidity. Survival rates were similar to those reported in our study. Despite that our study represents the first experience comparing HIPEC with curative and prophylactic intent respect to surgery alone, some major limitations should be evidenced.

First, all data were retrospectively collected, and hence, potential biases could derive from the study design. Second, it reports a single-center non-randomized experience with small sample size groups.

Thirdly, patients with uncontrolled severe infection and/or medical problems unrelated to malignancy were excluded from HIPEC. The selection of treatment results in uncontrollable biases.

Even so, we can conclude that in our experience, in selected patients with AGC, surgery plus HIPEC had a better OS and DFS with respect to patients treated with surgery alone.

## Conclusions

In conclusion, according to the results of the present study, patients with AGC undergoing surgery plus HIPEC, both with prophylactic and curative intent, had a better OS and DFS with respect to patients treated with surgery alone. Nevertheless, the role of CRS with HIPEC in AGC with macroscopic PC is still evolving and needs to be addressed in large multi-institutional randomized trials.

Moreover, some issues in the use of HIPEC as an adjuvant treatment in GC—choice of drug, dosage, duration of treatment, for which there is no consensus—are far to be resolved.

Widespread acceptance and adoption of prophylactic and curative HIPEC in AGC require a satisfactory answer to these issues.

## Declarations

All procedures followed were in accordance with the ethical standards of the responsible committee on human experimentation (institutional and national) and with the Helsinki Declaration of 1964 and later versions.
